# Reduced Susceptibility and Resistance to Vancomycin of Staphylococcus aureus: A Review of Global Incidence Patterns and Related Genetic Mechanisms

**DOI:** 10.7759/cureus.18925

**Published:** 2021-10-20

**Authors:** Susmita Unni, Tahseen J Siddiqui, Satesh Bidaisee

**Affiliations:** 1 Public Health, St. George’s University, St. George’s, GRD; 2 School of Medicine, St. George’s University, St. George’s, GRD

**Keywords:** cell wall thickening, mu3, mu50, vana gene, hvisa, heterogeneous vancomycin-intermediate staphylococcus aureus, vrsa, vancomycin-resistant staphylococcus aureus, visa, vancomycin-intermediate staphylococcus aureus

## Abstract

*Staphylococcus aureus* is a Gram-positive bacterium causing a wide range of infections ranging from cutaneous infections to endocarditis and bacteremia. Beta-lactamases such as penicillin and, subsequently, methicillin have been used in the treatment of *S. aureus infections*. With the emergence of methicillin-resistant *Staphylococcus aureus *(MRSA), vancomycin, a bacterial cell wall synthesis inhibitor, has been used as the treatment of choice for MRSA infections.

However, over the past few decades, there have been reports of reduced susceptibility and resistance of *S. aureus* to vancomycin globally, most recently from Michigan, United States, in July 2021. Based on the minimum inhibitory concentration (MIC) of the antibiotic against *S. aureus*, there are three strains of resistance, vancomycin-intermediate *Staphylococcus aureus* (VISA), vancomycin-resistant *Staphylococcus aureus* (VRSA), and heterogeneous vancomycin-intermediate *Staphylococcus aureus* (hVISA).

The increasing prevalence of VISA and VRSA infections is a cause of global concern. This qualitative review of peer-reviewed research publications aims to describe the cases of VISA and VRSA reported in the literature globally and summarizes the genetic mechanisms implicated in their resistance. The most common mechanism implicated in VRSA infections is the vanA operon, while cell wall thickening is responsible for VISA infections. This review aims to perform a global comparison between the MIC corresponding to the strength of resistance to vancomycin and the presence of the vanA operon. In this review, VISA and VRSA are noted to be most susceptible to quinupristin-dalfopristin and linezolid, respectively. Maintaining active systemic surveillance for such infections, employing strict infection control measures, and continuing to mitigate indiscriminate and irrational use of antibiotics are some of the actions that can be undertaken to reduce the incidence and transmission of VISA, VRSA, and hVISA infections worldwide.

## Introduction and background

*Staphylococcus aureus*, a Gram-positive bacterium, has been responsible for both community-acquired and hospital-acquired infections. This bacterium is found on the skin and the mucosal membranes of healthy individuals. It is a part of the normal human skin flora and is widely present in the environment [1]. Ranging from the skin to the bloodstream, *S. aureus* causes severe infections. In the 1940s, penicillin was the first antibiotic used to treat *S. aureus* infections [2]. Penicillin is a beta-lactam antibiotic that covalently binds to various penicillin-binding enzymes, known as penicillin-binding proteins. This leads to the inhibition of biosynthesis of the cell wall, causing a bactericidal effect on *S. aureus* [3]. *S. aureus *began producing an extracellular beta-lactamase (penicillinase) enzyme which inactivated the antibiotic through hydrolysis of the beta-lactam ring [3]. The adaptability of the bacteria to fight antibiotics through mutations and other mechanisms led to penicillin resistance. Widespread resistance to penicillin was first noticed in the 1950s [2]. Methicillin, a semi-synthetic beta-lactam antibiotic, was first used in the late 1950s as a treatment for the new penicillin-resistant *Staphylococcus aureus* (PRSA) infections (PRSA) [4]. This antibiotic covalently binds penicillin enzymes like carboxypeptidases and transpeptidases, which inhibits the synthesis of the bacterial cell wall [3]. Within a few years of using this treatment against PRSA infections, the first case of methicillin-resistant *Staphylococcus aureus* (MRSA) was reported [4]. By the 1970s, there was widespread resistance to this semi-synthetic group of penicillinase-resistant antimicrobial agents [2]. Further studies indicated that the resistance to both these classes of antibacterial agents was due to a low-affinity penicillin-binding protein (PBP) called the PBP2a [5]. The MRSA isolates were reported to contain a genetic element known as *SCCmec* within which a specific gene known as *mecA* was responsible for encoding PBP2a [6-8]. Beta-lactam antibiotics are unable to bind to PBP2a, leading to antibiotic resistance [5].

A new antibiotic was then needed to treat these infections that did not require attachment to the PBP2a site. Vancomycin, a glycopeptide, was first used to treat MRSA infections in a hospital setting in the late 1980s [4]. It functions by inhibiting cell wall synthesis of Gram-positive bacteria by attaching itself to the D-alanyl-D-alanine (D-ala-D-ala) terminus of the peptidoglycan cell wall [9]. This leads to a conformational change that prevents the precursor from attaching to the growing peptidoglycan chain, leading to cell wall decomposition and lysis of the bacteria [10]. It is currently the prevalent drug of choice for the treatment of severe MRSA infections. Vancomycin, and its structural relative teicoplanin, were the dominant drugs used historically to treat MRSA infections. However, in the 1980s, the first case of reduced susceptibility to teicoplanin was reported in Europe [4]. Vancomycin continued to be effective against MRSA infections.

The first case of reduced susceptibility of *S. aureus* to vancomycin was reported in Japan in 1997 [11]. Thereafter, various cases were reported in every continent except Oceania [12]. With the continued use of vancomycin, the first case of vancomycin-resistant *Staphylococcus aureus* (VRSA) was reported in the United States in Michigan in 2002, followed by cases in New York, New Jersey, and Delaware [13]. Further studies indicated that the origin of VISA was preceded by heterogeneous vancomycin-intermediate *Staphylococcus aureus* (hVISA) [14,15]. There were also cases of hVISA, vancomycin-intermediate *Staphylococcus aureus* (VISA), and VRSA infections reported from every continent.

This indicates that resistance to vancomycin, which is currently a highly reliable antibiotic for the treatment of MRSA infections, is a cause for global concern. This narrative review aims to identify and analyze the most common causes of resistance to vancomycin, compare the genetic mechanisms of VISA and VRSA infections, and their relationship with the strength of resistance against vancomycin, and determine which antibiotics VRSA and VISA are most susceptible to.

## Review

Methodology

Search Criteria

A qualitative analysis of peer-reviewed publications was conducted. The analysis focused on the genetic causes of VISA and VRSA infections. Because these infections have been reported worldwide and have been a source of public health concern, a qualitative analysis of the most common genetic cause of this resistance to vancomycin was reviewed. The literature search was performed via PubMed, Web of Sciences databases, and Google Scholar. The timeline set for the search was from January 1997 to September 2021 to track all cases since the first report in 1997 of reduced susceptibility of *S. aureus* to vancomycin. Global case reports were reviewed to provide a holistic view of the infections. The original data was gathered through published case reports presented by physicians, microbiologists, infectious disease specialists, and public health practitioners.

Inclusion and Exclusion Criteria

The cases were chosen based on the clinical report of the infections, analysis of the genetic causes, investigation into potential hospital and community exposure, and treatment performed for the infections. These factors would contribute toward the objective of the review to provide the incidence pattern and genetic mechanism of the infections. Cases that did not include genetic analysis of the strain could not contribute toward the aim of reviewing genetic mechanisms and were therefore excluded from the review.

Data Extraction

From each study, the genetic components of the strains, the minimum inhibitory concentration (MIC) of vancomycin, and the antibiotic resistance and susceptibility data were extracted. Additionally, the year and geographical location of the infection were extracted as well.

Data Analysis

The data were divided into groups of VRSA and VISA infections. The data were further divided into subgroups of continents. A comparison between the genetic causes, MIC, and antibiotic susceptibility was performed. In addition, a comparison of the cause of VRSA infection to its respective MIC was performed as well. An intercontinental analysis of the most common antibiotics effective against these infections was also performed. An analysis of the etiology, MIC, and treatment sensitivity can help curtail the incidence and spread of these antibacterial strains. Because this group of *S. aureus* strains that are building resistance to vancomycin is relatively new, there are significant limitations to this review. There could potentially be a significant number of unreported cases, especially in developing countries. The resources to identify and treat these infections could be limited by knowledge, medical infrastructure, and economic constraints. Even if the cases were treated, there could be a lack of publications to track these infections. Due to the different sensitivity profiles of the infections, a primary treatment often could not be identified.

Discussion

There are three currently identified patterns of *S. aureus* resistance to the glycopeptide antibiotic vancomycin. This is determined by the concentration of the antibacterial agent required to inhibit the growth of the bacterium and is termed the MIC. When *S. aureus* is sensitive to vancomycin, it is termed vancomycin-sensitive *S. aureus* (VSSA) and has an MIC of ≤2 μg/mL [16]. The Center for Disease and Infection Control (CDC) has determined the concentrations for the classification of these strains with reduced sensitivity to vancomycin. The first is VISA with an MIC of ≥8 μg/mL. The second is VRSA with an MIC of ≥16 μg/mL. There is also a third resistant strain which is determined to be the precursor of VISA [17], the hVISA strain. It has heterogeneous qualities with various degrees of resistance to vancomycin and subpopulations of VISA daughter cells [17].

In 1997, a Japanese hospital reported its first case of *S. aureus* infection with reduced susceptibility to vancomycin, which came to be termed as VISA [11]. Subsequently, cases of VISA were reported in the United States, Europe, and Asia [2]. The first case of VRSA was reported in Michigan, United States, in 2002 [13]. As of 2017, a total of 14 cases of VRSA were reported in the United States [4], with no further cases reported over the next three years. However, in 2021, the 15th case of VRSA was reported in Michigan [18]. Shariati et al. reported that since 2010, there has been an increase in the incidence of reported VISA, VRSA, and hVISA infections by 3.6, 2.0, and 1.3 folds, respectively, compared to previous years [12]. As of 2020, there is a high prevalence of VRSA (3.6%) and hVISA (5.2%) in the United States, while Asian countries reported a high prevalence of VISA (2.1%) [12]. Due to the instability with concentration susceptibility and resistance to vancomycin in hVISA, screening is difficult [17]. Therefore, this review focused on genetic factors leading to VRSA and VISA infections.

There are various causes of *S. aureus* resistance to vancomycin. One study described the most common cause of VRSA infections as resistance mediated by the vanA operon, which is also a cause of vancomycin-resistant *Enterococcus faecalis*. For *S. aureus* to develop resistance via this mechanism, it requires the D-ala-D-ala terminal to be replaced by D-ala-D-lactate [4]. This is mediated by the vanA operon found on the transposon Tn1546 and is carried by Inc18-like plasmid to the mutation site [9]. The enzymes encoded on the transposon are responsible for the conversion of D-ala-D-ala to D-ala-D-lactate, thereby decreasing the affinity of vancomycin by a factor of 1,000 compared to the normal cell wall [4]. In attempts to understand the emergence of the vanA operon in *S. aureus*, there were concerns of conjugate transfer of the vanA operon from *E. faecalis* to *S. aureus* [9]. This led to in-vitro studies that indicated the presence of pSK-41-like plasmid that could carry the Inc18-like vanA plasmids from enterococci to *S. aureus*. Other genetic mutations were noted to cause vanA transfer from *E. faecalis* to *S. aureus*. However, the Inc18-like plasmid appears to be the most common cause of this transfer. In the United States, the Inc18-like plasmid was present in 8/15 VRSA cases [10]. The United States also designates names for their strains based on their pulse-field gel electrophoresis (PFGE) patterns. There are two strains named USA 100 and ST5, which belong to the clonal complex 5 (CC5) and clonal complex 8 (CC8) strains, respectively, and are commonly derived from healthcare-associated MRSA infections [19].

Another common cause of VRSA and VISA infections is the cell wall thickening of *S. aureus* bacteria. The strains with increased cell wall thickening are Mu50 and Mu3. Mu3 is the heterogeneous strain of Mu50 and is therefore associated with hVISA infections with an MIC of ≤4 μg/mL [20]. Mu50 is associated with vancomycin resistance with an MIC of ≥8 μg/mL, and it has double the cell wall thickness compared to Mu3 [21]. Due to its increased cell wall thickness, there is affinity trapping of vancomycin molecules on the outer membrane of the peptidoglycan cell wall, and, thus, the antibiotic cannot reach the PBP2 and PBP2’ binding sites on the cytoplasmic membrane to decrease cross-linking and interrupt cell wall biosynthesis, which leads to resistance [21]. Mu50 and Mu3 strains of VISA and hVISA do not contain any of the enterococcal van genes and, therefore, have been shown to develop resistance without transfer of van genes from VRE infections [21]. However, studies indicate that prolonged and repeated use of vancomycin can lead to increased cell wall thickening, which makes the bacterial strain more impermeable to the antibiotic.

The first case of VRSA infection was reported in the United States in 2002 [22,23]. The most common cause of *S. aureus* resistance to vancomycin reported in the United States is the presence of a vanA gene [22-27]. All except one strain belong to the CC5 group, which is associated with hospital-acquired MRSA infections [22-29]. Only the strain from Delaware, reported in 2002, was found to have the clonal complex 30 (CC30), which belongs to the group of the community-acquired strains of MRSA infection [18]. In Asia, strains from North India did not detect vanA genes [30]. The MICs were between 16 and 64 μg/mL [30]. Furthermore, the soft tissue isolate from Tehran also did not detect the presence of the vanA gene. The MIC for this isolate was 64 μg/mL. The Nigerian VRSA isolate from a patient’s surgical infection site also did not detect vanA or vanB genes, and the MIC for this isolate was 16 μg/mL.

**Figure 1:**
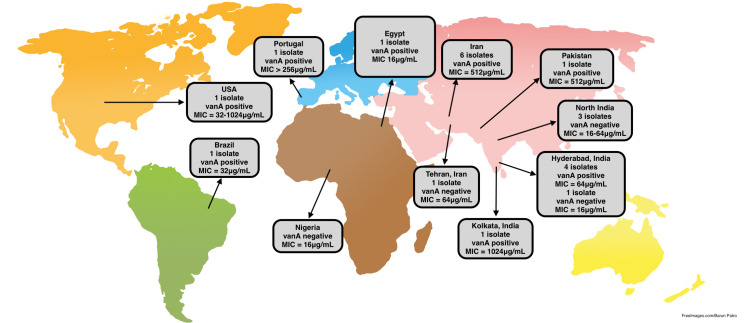
VRSA isolates with MIC to vancomycin related to the presence/absence of the vanA gene. VRSA = vancomycin-resistant Staphylococcus aureus; MIC = minimum inhibitory concentration

An analysis of different countries from various continents that reported cases of resistance provides evidence that VRSA infections are most commonly due to the vanA operon gene mutation in the presence of VRE infections, MRSA infections, or both (Table 1). There are some exceptions to these observations, as described above [30]. There is an observable difference between the MIC of vancomycin to the strain that does and does not have vanA or vanB genes. Most strains with vanA genes have MICs ranging between 128 μg/mL and 1,024 μg/mL (Figure 1). The strains without vanA genes have MICs ranging between 16 and 64 μg/mL (Figure 1). This indicates that the presence of the vanA gene can cause a stronger resistance to vancomycin. Further research and analysis are needed to determine the strength of the vanA gene resistance to vancomycin in *S. aureus*. The resistance of the vanA-negative strains is hypothesized to be due to cell wall thickening, the presence of MRSA/VRE infections, or prolonged exposure to vancomycin.

**Table 1:** Reported cases of VRSA with genetic analysis, MIC, resistant, and susceptible antimicrobial agents. AMK = amikacin; AMP = ampicillin; AMP-SUL = ampicillin-sulbactam; AMX = amoxicillin; AMX-CLV = amoxicillin-clavulunate; ARB = arbekacin; CAZ = ceftazidime; CEX = cephalexin; CFPM = cefepime; CFZ = cefazolin; CHL = chloramphenicol; CIP = ciprofloxacin; CLI = clindamycin; CLR = clarithromycin; CMX = co-trimoxazole; CRO = ceftriaxone; CTX = cefotaxime; CXM = cefuroxime; DAP = daptomycin; DOX = doxycycline; ERY = erythromycin; FOX = cefoxitin; FQ = fluoroquinolone; FUS = fusidic acid; GEN = gentamycin; KAN = kanamycin; LVX = levofloxacin; LZD = linezolid; MAC = macrolide; MET = methicillin, MIN = minocycline; MOX = moxifloxacin; MUP = mupirocin, NB = novobiocin; NIT = nitrofurantoin; OXY = oxacillin; PEN = penicillin; PIS = pistinamycin; QD = quinupristin/dalfopristin; RIF = rifampin; SCF = cefoperazone-sulbactam; TEI = teicoplanin; TET = tetracycline; TGC = tigecycline; TMP-SMX = trimethoprim-sulphamethoxazole; TOB = tobramycin; VRSA = vancomycin-resistant *Staphylococcus aureus*; MIC = minimum inhibitory concentration

Year	Location	Site	Cause of resistance	MIC (μg/mL)	Antimicrobial agent: Resistant	Antimicrobial agent: Susceptible	Ref.
United States							
2002	Michigan	Toe amputation wound	vanA plasmid with Tn1546 USA100	32	-	CHL, LZD, QD, TMP-SMX	[22,23]
2002	Pennsylvania	Chronic foot ulcer	mecA, vanA plasmid with Tn1546	32	OXY	CHL, LZD, MIN, QD, RIF, TMP-SMX	[24]
2004	New York	Indwelling nephrostomy tube in patient	vanA gene USA800	32–128	AMK, FQ, MAC, PEN, TET	CHL, LZD, RIF, TMP-SMX	[25]
2005	Michigan	Toe wound	vanA USA100	256	CLI, ERY, OXY, LVX, TEI	DAP, DOX, GEN, LZD, QD, RIF	[26]
2005	Michigan	Plantar wound	vanA plasmid with Tn1546 ST5	1,024	CLI, ERY, OXY, LVX, TEI	DAP, DOX, GEN, LZD, QD, RIF	[26]
2006	Michigan	Triceps wound	vanA Inc-18 like plasmid	512	CLI, ERY, OXY, LVX, TEI	DAP, DOX, GEN, LZD, QD, RIF	[26]
2007	Michigan	Plantar foot wound	vanA Inc18-like plasmid	1,024	TMP-SMX	DAP, LZD, QD, RIF, TET, TGC, CHL	[27]
2007	Michigan	Plantar foot wound	vanA	1,024	TMP-SMX	DAP, LIN, QD, RIF, TET, TGC	[27]
2009	Michigan	Plantar foot wound	USA100 CC5	-	-	-	[28]
2010	Delaware	Wound drainage	USA100 CC5	-	-	-	[28]
2010	Delaware	Vaginal swab	CC5	-	-	-	[28]
2012	Delaware	Foot wound	USA100 CC30 (community-acquired) vanA Inc-18-like plasmid	256	CTX, CLI, ERY, LVX	TMP-SMX	[18]
2015	Delaware	Chronic toe wounds	vanA USA100 CC5	512	-	-	[29]
Asia							
2002–2005	Northern India	Pus-surgery ward	(-) vanA,(-) vanB	64	PEN, OXY, GEN, TOB, AMK, CIP, CHL, ERY, TMP-SMX, SCF	TET, NIT	[30]
		Pus-skin wound		32	PEN, OXY, GEN, TOB, AMK, CIP, CHL, ERY, TMP-SMX, NIT	TET, SCF	
		Pus-orthopedic outpatient		16*	PEN, OXY, GEN, TOB, AMK, CIP, CHL, ERY, NIT	CHL, TMP-SMX, SCF	
2005	Kolkata (India)	Pus-outpatient	vanA vanHax-analogous to Tn1546-like plasmid	1024	AMX, AMP, CFPM, CTX, CEX, CHL, CLI, ERY, RIF, MET	GEN, CIP	[31]
2005	Tehran (Iran)	Soft tissue wound	(-) vanA, (-) vanB	64	CTX, CRO, ceftizoxime	TEI, TET, LZD, DOX, ERY, CLI, CFZ	[32]
2005	Tehran (Iran)	Post-heart surgery wound	vanA	512	TEI, CFZ, CLI, CRO, CTX, ERY	TET, LZD, DOX	
2008	Hyderabad (India)	Wound swab, urine, ear swab, blood, throat swab	6/7 isolates (+) vanA blood (-) vanA	16-64	CAZ, RIF	TET, CHL, CLR	[33]
2010	Karachi (Pakistan)	Diabetic foot ulcer	vanA with Tn1546	512	-	-	[34]
2012	Shiraz (Iran)	NICU patients, surgical wounds	vanA, vanB		AMP, PEN, AMX, TET, ERY, OXY, CLI, RIF, LZD, TEI, QD	CIP	[35]
2012	Iran	Diabetic foot ulcer	vanA with Tn1546	512	-	-	[36]
2017	Tehran (Iran)	Troat swab, bronchial aspirate, wound, blood	VanA	512 512 64 64	PEN, CRO, KAN, CIP, CLI, TET, ERY, TEU, AMK, TOB, GEN, TMP-SMX, RIF	LZD	[37]
2021	South Korea	Post-liposuction infection	vanA		-	CLI, MOX	[38]
Africa							
2013	Egypt	Skin swab	vanA gene via conjugate transfer	>/=16	PEN, AMX-CLV, AMP-SUL	CIP, AMK	[39]
2018	Nigeria	Surgical infection site	Nuc gene vanA, (-) vanB	16	AMX, ERY, TET, GEN, CMX, CHL, FUS, NB	-	[40]
South America							
2012	Brazil	Bloodstream	vanA gene with Tn1546-like plasmid and Inc18-like plasmid	32	ERY, CLI, CIP, GEN, TMP-SMX	-	[41]
Europe							
2013	Portugal	Toe amputation wound	vanA gene	>256	ERY, CLI, GEN, CIP	CMX, TET, TGC, LZD, DAP, QD, FUS, CHL, RIF, MUP	[42]

**Figure 2:**
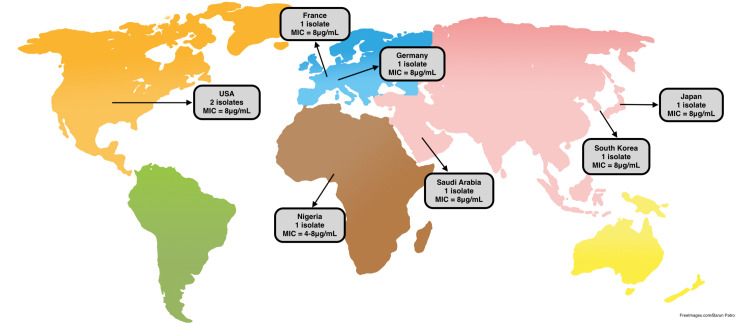
VISA isolates with MIC to vancomycin related to the presence/absence of the vanA gene. VISA = vancomycin-intermediate *Staphylococcus aureus*; MIC = minimum inhibitory concentration

Similarly, the data for VISA infections from different countries indicates that cell wall thickening is the most common cause of decreased efficacy of vancomycin to *S. aureus* infections (Table 2). The MIC is 4-8 μg/mL (Figure 2). Comparing the genetic causes of VRSA to VISA infections, it is noted that none of the reviewed VISA infections presented with vanA or vanB genes. Each of them appeared to have an increased cell wall thickness, which included the Mu50 strain that had a seven-fold increase in thickness compared to normal strains, and the Mu3 strain that had a few extra layers of thickness compared to normal strains [11,43-45].

**Table 2:** Reported cases of VISA with genetic analysis, MIC, antibiotic resistance, and susceptibility. AMK = amikacin; AMP = ampicillin; AMP-SUL = ampicillin-sulbactam; AMX = amoxicillin; AMX-CLV = amoxicillin-clavulunate; ARB = arbekacin; CAZ = ceftazidime; CEX = cephalexin; CFPM = cefepime; CFZ = cefazolin; CHL = chloramphenicol; CIP = ciprofloxacin; CLI = clindamycin; CLR = clarithromycin; CMX = co-trimoxazole; CRO = ceftriaxone; CTX = cefotaxime; CXM = cefuroxime; DAP = daptomycin; DOX = doxycycline; ERY = erythromycin; FOX = cefoxitin; FQ = fluoroquinolone; FUS = fusidic acid; GEN = gentamycin; KAN = kanamycin; LVX = levofloxacin; LZD = linezolid; MAC = macrolide; MET = methicillin; MIN = minocycline; MOX = moxifloxacin; MUP = mupirocin; NB = novobiocin; NIT = nitrofurantoin; OXY = oxacillin; PEN = penicillin; PIS = pistinamycin; QD = quinupristin/dalfopristin; RIF = rifampin; SCF = cefoperazone-sulbactam; TEI = teicoplanin; TET = tetracycline; TGC = tigecycline; TMP-SMX = trimethoprim-sulphamethoxazole; TOB = tobramycin; MRSA = methicillin-resistant *Staphylococcus aureus*; VISA = vancomycin-intermediate *Staphylococcus aureus*; MIC = minimum inhibitory concentration

Year	Location	Site	Cause of resistance	MIC (μg/mL)	Resistant antibiotics	Susceptible antibiotics	Ref.
United States							
1997	Michigan	Peritoneal catheter	Increased cell wall thickness	8	PEN, OXY, CIP, ERY, CLI, GEN, TEI	CHL, QD, TET, TMP-SMX, RIF	[13]
1997	New Jersey	Bloodstream	Increased cell wall thickness	7	PEN, OXY, CIP, ERY, CLI, RIF	CHL, QD, TET, TMP-SMX, GEN, TEI	[13]
Asia							
1997	Japan	Sternal surgical incision site	Mu50 3-fold increase in cell wall thickness	8	-	ARB, AMP-SUL	[11]
2000	Korea	Bloodstream	Cell wall 2-3 times thicker than Mu3 (possibly Mu50)	8	TEI, OXY, CLI, ERY, CIP, RIF, GEN, QD	-	[43]
2020	Saudi Arabia	Bloodstream	Mu3-like profile	8	PEN, MET, CIP, LVX, FUS	RIF, TGC, LZD	[44]
Africa							
2018	Nigeria	-	vanA, vanB (-) Possibly d/t thickened cell wall	4-8	GEN, CHL, FUS, NB	-	[40]
Europe							
1998	France	Bloodstream	vanA, vanB (-) restriction gel pattern indicates derived from MRSA isolate	8	TEI, AMK	PIS, TMP-SMX, QD	[45]
1999	Germany	-	Two-fold increase in cell wall thickening compared to Mu50 7-fold increase in cell wall thickening	8	-	QD	[46]

**Figure 3:**
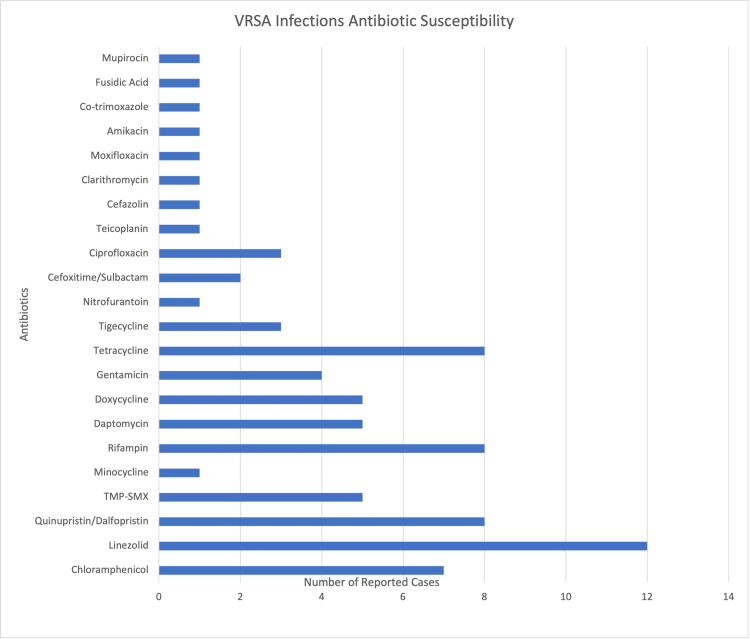
Number of cases of VRSA with antibiotic susceptibilities. VRSA infections worldwide appear to be most susceptible to linezolid. VRSA = vancomycin-resistant *Staphylococcus aureus*

**Figure 4:**
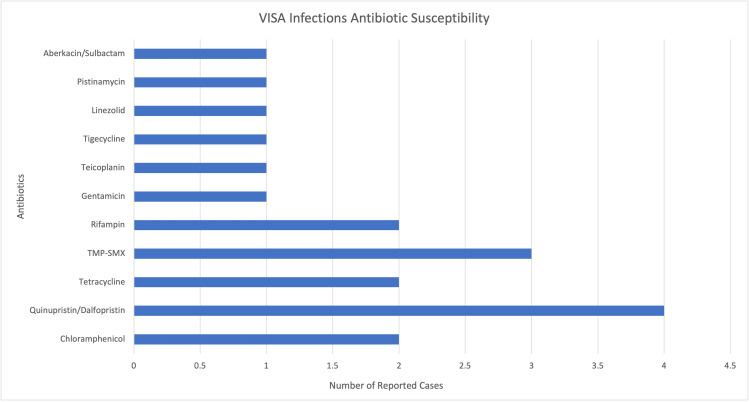
Number of cases of VISA with antibiotic susceptibilities. VISA infections worldwide appear to be most susceptible to quinupristin/dalfopristin. VISA = vancomycin-intermediate *Staphylococcus aureus*

An analysis of the antibiotic susceptibility from the cases reviewed indicates that VRSA infections were most susceptible to linezolid (Figure 3) [22-42]. Similarly, VISA infections were most susceptible to quinupristin/dalfopristin (Figure 4) [11,43-46]. However, as recommended by the CDC, susceptibility testing is extremely important before implementing a focused treatment plan [2]. VISA and VRSA are reportable infections. In the United States, CDC has issued guidelines to inpatient and outpatient healthcare facilities to notify local and state authorities of identified cases for further analysis [2].

There are several limitations to this study as all the reported infections worldwide did not have a genetic analysis performed that identified the cause of resistance. There are limited resources available worldwide to perform confirmatory tests for these infections. Additionally, there is still a lack of complete understanding regarding the acquired resistance of *S. aureus* to vancomycin.

## Conclusions

Vancomycin is the preferred treatment available against MRSA infections and remains the treatment of choice globally. The development of resistance to vancomycin is a threat to global public health. There are currently limited cases of VRSA and VISA infections reported. However, there is at least a two-fold increase in the number of reported cases in the past 10 years, which is of major concern. The vanA operon is the most common cause of VRSA infections worldwide, and increased cell wall thickness is the most common cause of VISA infections. The irrational use of antibiotics in hospitals and the availability of antibiotics over the counter in certain countries will likely aggravate this problem. Therefore, it is important to maintain active systemic surveillance of these infections.
